# Effect of early oxygen therapy and antiviral treatment on disease progression in patients with COVID-19: A retrospective study of medical charts in China

**DOI:** 10.1371/journal.pntd.0009051

**Published:** 2021-01-06

**Authors:** Lu Long, Liang Wu, Lang Chen, Daixing Zhou, Hongyu Wu, Donghao Lu, Honglin Li, Xiaoxia Duan, Yutong Han, Xianzhi Li, Qiuxia Wang, Jing Zhang

**Affiliations:** 1 Department of Epidemiology and Biostatistics, West China School of Public Health and West China Fourth Hospital, Sichuan University, Chengdu, China; 2 Channing Division of Network Medicine, Department of Medicine, Brigham and Women’s Hospital, and Harvard Medical School, Boston, Massachusetts, United States of America; 3 Department and Institute of Infectious Disease, Tongji Hospital, Tongji Medical College, Huazhong University of Science and Technology, Wuhan, China; 4 Department of Radiology, Tongji Hospital, Tongji Medical College, Huazhong University of Science and Technology, Wuhan, China; 5 Department of Emergency medicine, Tongji hospital, Tongji Medical College, Huazhong University of Science and Technology, Wuhan, Hubei, China; 6 Department of Medical Epidemiology and Biostatistics, Karolinska Institute, Stockholm, Sweden; 7 Department of Epidemiology, Harvard T.H. Chan School of Public Health, Boston, Massachusetts, United States of America; 8 Department of Pediatric Surgery, Tongji Hospital, Tongji Medical College, Huazhong University of Science and Technology, Wuhan, China; Institute for Disease Modeling, UNITED STATES

## Abstract

**Background:**

Until now, no antiviral treatment has been proven to be effective for the coronavirus disease 2019 (COVID-19). The timing of oxygen therapy was considered to have a great influence on the symptomatic relief of hypoxemia and seeking medical intervention, especially in situations with insufficient medical resources, but the evidence on the timing of oxygen therapy is limited.

**Methods and findings:**

Medical charts review was carried out to collect the data of hospitalized patients with COVID-19 infection confirmed in Tongji hospital, Wuhan from 30^th^ December 2019 to 8^th^ March 2020. In this study, the appropriate timing of oxygen therapy and risk factors associated with severe and fatal illness were identified and the effectiveness of antivirus on disease progression was assessed. Among 1362 patients, the prevalence of hypoxia symptoms was significantly higher in those patients with severe and fatal illness than in those with less severe disease. The onset of hypoxia symptoms was most common in the second to third week after symptom onset, and patients with critical and fatal illness experienced these symptoms earlier than those with mild and severe illness. In multivariable analyses, the risk of death increased significantly when oxygen therapy was started more than 2 days after hypoxia symptoms onset among critical patients (OR, 1.92; 95%CI, 1.20 to 3.10). Compared to the critically ill patients without IFN-a, the patients who were treated with IFN-a had a lower mortality (OR, 0.60; 95%CI, 0.39 to 0.91).

**Conclusions:**

Early initiation of oxygen therapy was associated with lower mortality among critical patients. This study highlighted the importance of early oxygen therapy after the onset of hypoxia symptoms. Our results also lend support to potentially beneficial effects of IFNα on critical illness.

## Author summary

Until now, no antiviral treatment has been proven to be effective for the coronavirus disease 2019 (COVID-19). The timing of oxygen therapy, which has rarely been mentioned in the current literature, has a great influence on the symptomatic relief of hypoxemia and seeking medical intervention, especially in situations with limited medical resources. It is worthwhile to find clues from the exploratory use of oxygen and antiviral drugs in Wuhan at the beginning of the COVID-29 pandemic. Therefore, in this study, a retrospective review was carried out to identify the risk factors associated with a severe and fatal illness, including the use of antivirus and timing of oxygen therapy. The main differences in the clinical features of patients with different severities of COVID-19 were described and the risk factors were explored to expand the current recommendations for high-risk groups. Initiation of oxygen treatment less than 2 days after onset after onset of hypoxia symptoms and the use of IFN-a among critically ill patients were significantly associated with lower risk of COVID-19 mortality. Our study highlighted the importance of early oxygen therapy and lend support to the potentially beneficial effects of IFNα on critical illness.

## Introduction

Coronavirus disease 2019 (COVID-19) is caused by a newly emergent coronavirus closely linked to severe acute respiratory syndrome (SARS) virus; COVID-19 can be complicated by ARDS, sepsis and septic shock, and multiorgan failure and is associated with ICU admission and high mortality [1–3]. Primary care doctors are often overwhelmed, with a large number of patients with COVID-19 seeking medical attention for the fear of severe respiratory complications and death.

Until now, no antiviral treatment has been proven to be effective against the virus [4]. The treatment of COVID-19 is symptomatic, and oxygen therapy is recommended by the World Health Organization (WHO) for patients with respiratory distress, hypoxemia, or shock, with a target SpO2 > 94% currently [5]. Timely oxygen therapy, which allows greater levels of oxygen to pass through the thickened and inflamed lung tissue into the bloodstream earlier, can effectively treat hypoxemia [6]. The timing of oxygen therapy, which has rarely been mentioned in the current literature, has a great influence on the symptomatic relief of hypoxemia and seeking medical intervention, especially in situations with limited medical resources.

In this context, it is worthwhile to find clues from the exploratory use of oxygen and antiviral drugs in Wuhan at the beginning of the COVID-29 pandemic. Therefore, a retrospective review of medical charts was carried out to identify the appropriate timing of oxygen therapy, the risk factors associated with a severe and fatal illness, and the effectiveness of antivirals that may be used for preventing disease progression in patients with COVID-19.

## Methods

### Ethics statement

This study was approved by the Tongji Hospital Ethics Committee, and due to the nature of the retrospective chart review, the need for informed consent from individual patients was waived.

### Data sources

**Fig 1:**
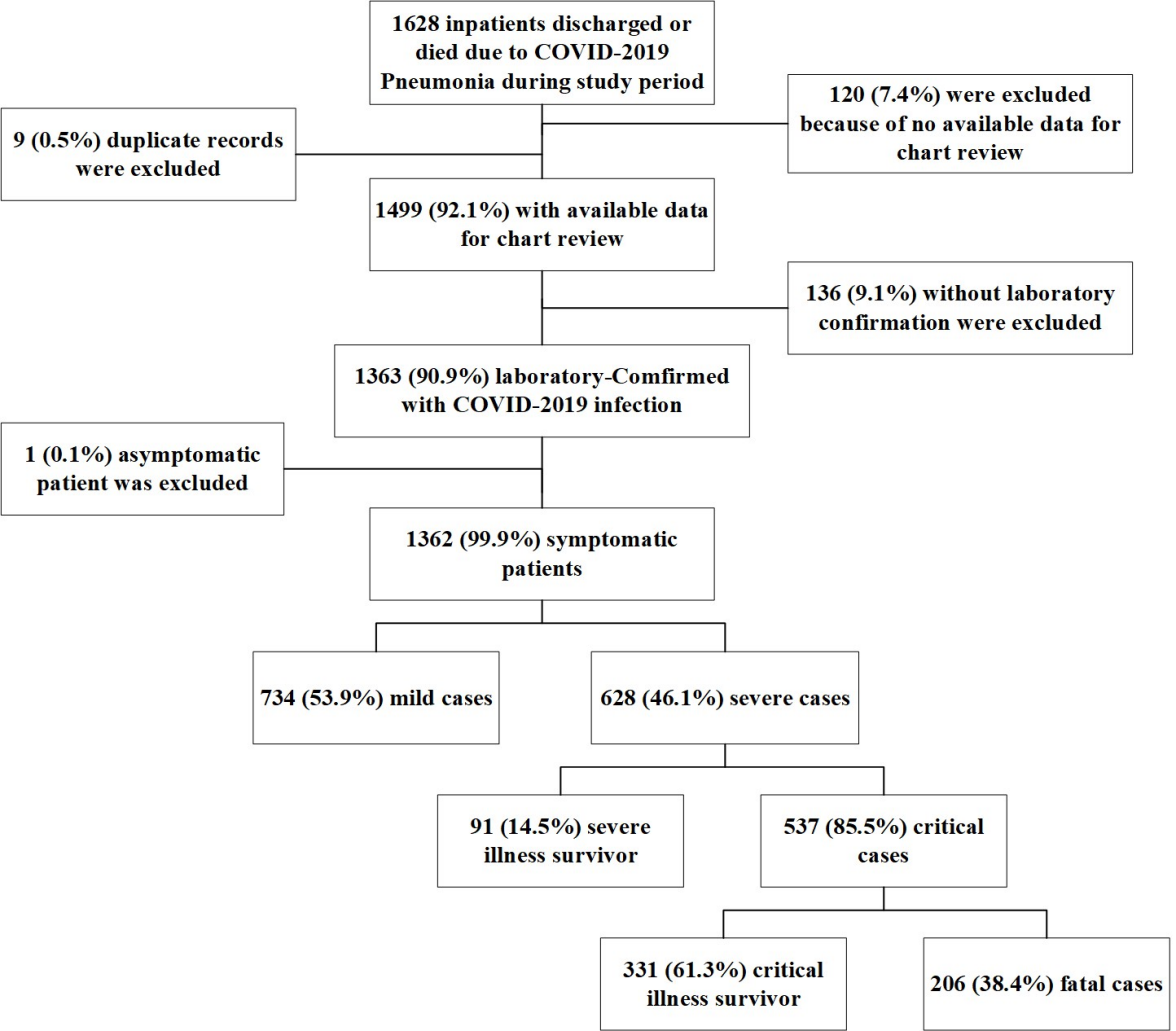
Flow chart of enrolment of 1362 confirmed COVID-2019 patients during the study period (from 30^th^ December 2019 to 8^th^ March 2020).

This retrospective observational study was performed at Tongji Hospital, which was urgently reconstructed and has been assigned by the Chinese government as a designed hospital for severely or critically ill patients with COVID-19. A total of 1362 patients with laboratory-confirmed COVID-19 infection admitted to Tongji Hospital from 30^th^ December 2019 to 8^th^ March 2020 were enrolled. The main components of the patient enrollment process are shown in Fig 1.

### Case definitions

COVID-19 was confirmed by detecting SARS-CoV-2 RNA in throat swab samples. The real-time RT-PCR assay was conducted using a SARS-CoV-2 nucleic acid detection kit according to the manufacturer’s protocol (Shanghai Biogerm Medical Technology Company).

All patients diagnosed with COVID-19 were classified as having mild, severe, or critical illness according to the Guidance for Corona Virus Disease 2019 (7th edition) released by the National Health Commission of China [7]. Mild illness was defined an uncomplicated upper respiratory tract viral infection, and with or without radiographic evidence of pneumonia. Serious illness was defined if satisfying at least one of the following items: (1) breathing rate ≥30/min; (2) pulse oximeter oxygen saturation (SpO2) ≤93% at rest; or (3) ratio of partial pressure of arterial oxygen (PaO2) to fraction of inspired oxygen (FiO2) ≤ 300 mmHg (1 mmHg = 0.133 kPa). Critical illness was defined if satisfying at least one of the following items: (1) respiratory failure occurred and patient received mechanical ventilation; (2) shock; or (3) failure of other organs and care received in the ICU.

### Data collection

The medical records of patients were analyzed by our research team, which was trained before the study began. We developed data abstraction forms based on previous research and our clinical experience. Medical record number, the course of disease onset, first symptom, treatment before admission, medical history, symptoms, signs, the onset time of all symptoms (which were searched from the first course), daily progress notes, and discharge record were included in the questionnaires. We double-entered the data using Epidata 3.1 software. Then, after checking the data and logicality, the unqualified data were rejected. If core data were missing, our coordinators would log in to the electronic medical record system for verification and contacted the doctor in charge of the patients when necessary. Other information, including demographics, epidemiological characteristics, laboratory test results, medicine use and oxygen therapy after admission, was directly obtained from electronic medical records.

### Antivirus treatment and oxygen therapy

The use of antiviral drugs in Tongji Hospital is described below.

1. Interferon-alpha (IFN-α): 5 million U or equivalent dose per time for adults, 2 ml injection of sterile water, twice daily atomization inhalation.
2. Lopinavir/ritonavir (LPV/r): 200 mg/50 mg/capsule for adults, 2 capsules per time, twice a day, for no more than 10 days.
3. Ribavirin: 500 mg/time, 2–3 times a day, for no more than 10 days.
4. Chloroquine or hydroxychloroquine (CQ or HCQ): for patients over 50 kg, 500 mg every time, twice a day, for no more than 7 days of treatment; for patients less than 50 kg, 500 mg every time for day 1–2, twice a day, 500 mg every time for day 3–7, once a day.
5. Arbidol; 200 mg, 3 times a day, for no more than 10 days.

Oxygen therapy could be started at a flow rate of 5 L/min, and the target oxygen saturation was indicated by a pulse oxygen saturation ≥90% in nonpregnant adult patients, ≥92–95% in pregnant patients, and ≥94% in patients who were critically ill with severe respiratory distress, shock, or coma. If standard oxygen therapy failed, mechanical ventilation was considered; high flow nasal catheter oxygen or noninvasive ventilation (for example, bilevel positive airway pressure mode) could also be used. If no improvement was seen within one hour of noninvasive mechanical ventilation, invasive mechanical ventilation was used. Experienced experts could recommend extracorporeal membrane oxygenation (ECMO) according to their evaluation of the patient’s condition.

### Statistical analysis

Descriptive statistics included frequency analysis for categorical variables, means and standard deviations for normally distributed continuous variables, or medians and interquartile ranges for continuous variables that were not normally distributed. Feeling breathless was described in different ways including breath shortness, dyspnea and chest tightness. These three symptoms were grouped as hypoxia symptoms.

Multivariate logistic regression with a backward-forward stepwise method was used to identify risk factors associated with subsequent development of severe illness or death, and odds ratios and 95% confidence intervals were calculated. We excluded from the models the variables for mechanical ventilation and ECMO treatment to avoid false inferences of causation due to reverse causation. Relationships between age and comorbidities were evaluated by Spearman's correlation analysis to determine the need to account for collinearity in the multivariate analyses. To identify the oxygen therapy initiation associated with the length of invasive mechanical ventilation and illness severity, only case patients who reported hypoxia symptoms and received oxygen therapy were included in the analysis. Optimal cut-off values of continuous variables in our study were determined based on the receiver-operating characteristic (ROC) curves.

To better understand the causal effect of antiviral treatment on the development of severe illness, we considered including patients who received antiviral treatment before progression to severe illness and excluding critical cases, because most of them were serious patients and had already received antiviral treatment upon admission. To assess the effectiveness of antiviral treatment among critical patients, we compared antiviral treatment in critical illness survivors and death.

Statistical analysis was performed with R Statistical Software (Foundation form Statistical Computing, Vienna, Austria). For all analyses, probabilities were two-tailed, and a *P* value of <0.05 was considered significant.

## Results

A total of 1362 confirmed COVID-19 patients were included in our study, including 206 (15.1%) who died and 1156 (84.9%) who were discharged from the hospital. Of the patients discharged from the hospital, 628 (54.3%) had severe illness, including 331 (28.6%) patients who developed critical illness (**Fig 1**).

**Fig 2:**
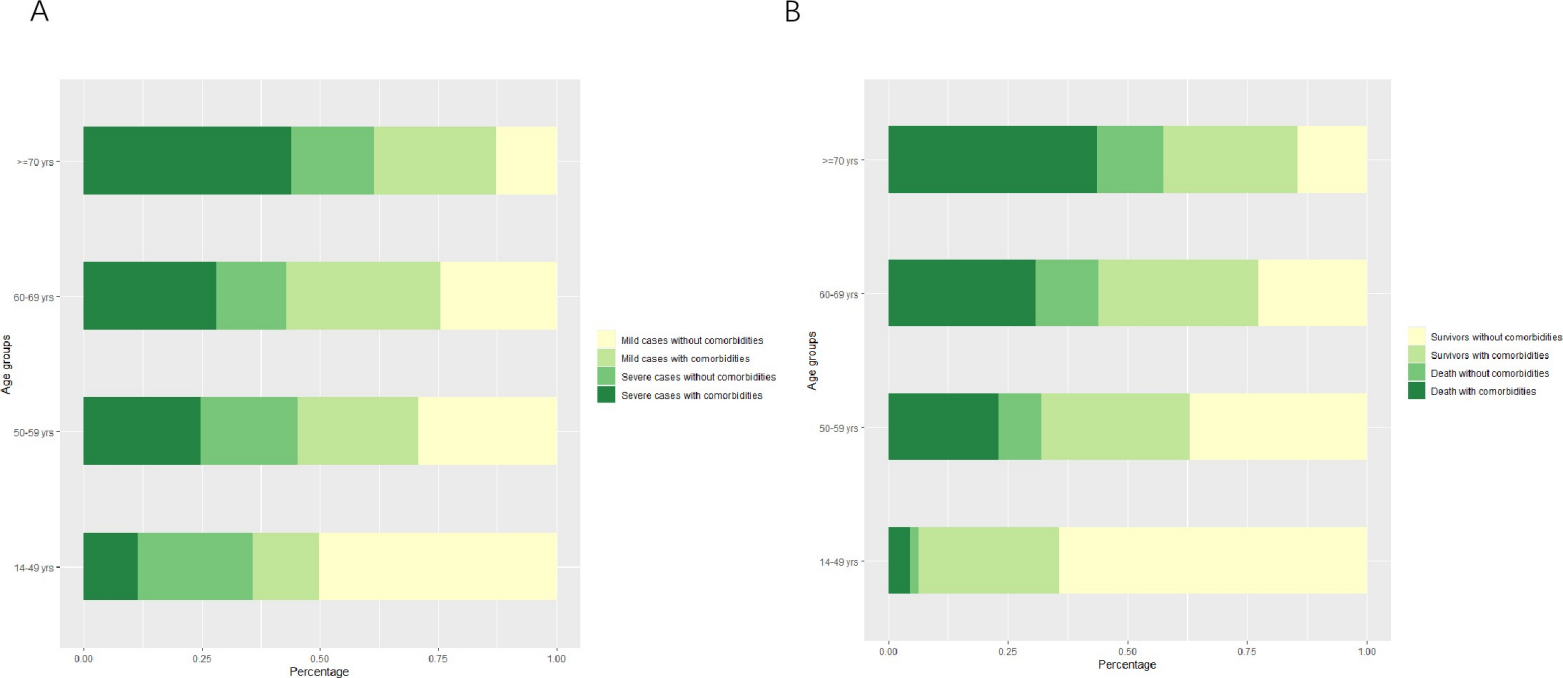
Percentages of patients under different chronic medical conditions and disease severity in different age groups. A. mild and severe cases with or without comorbidities. B. critical and fatal cases with or without comorbidities.

The median age of the patients was 62 years (interquartile range, IQR 49–69), and 50.3% were male (**Table 1**). The median age of the patients who died was 70 years, which was older than that of the patients who survived (*P* value <0.001). The number of fatal cases increased with increasing age, from 7 (3.4%) among patients 14–49 years of age to 103 (50%) among those over 70 years of age. Of the 1362 total patients, 704 (51.7%) had comorbidities; the most common were hypertension (30.3%), diabetes (14.2), and coronary disease (7.6%) (**Table 1**). The proportion of severe patients with comorbidities increased with age. Among patients over 70 years old, death with complications accounted for the largest proportion. Older patients with comorbidities were more likely to develop severe and fatal illness than younger patients (both *P* value <0.001) (**Fig 2 and** S1 Data).

**Table 1:** Demographic characteristics and clinical symptoms of 1362 inpatients with COVID-2019 in Wuhan, China. Figures are numbers (percentages) unless stated otherwise.

Characteristic	All case patients (n = 1362)	Patients with mild illness (n = 734)	Patients with severe illness			
			Subtotal (n = 628)	Survivors of severe illness (n = 91)	Survivors of critical illness (n = 331)	Death (n = 206)
Male	685 (50.3)	336 (45.8)	349 (55.6)	43 (47.3)	170 (51.4)	136 (66.0)
Median(IQR) age(year)	62 (49–69)	59 (45–67)	65 (52–72)	64 (55–71)	58 (45–69)	70 (63–78)
Age group(year)						
14–49	357 (26.2)	229 (31.2)	128 (20.4)	16 (17.6)	105 (31.7)	7 (3.4)
50–59	263 (19.3)	144 (19.6)	119 (18.9)	19 (20.9)	68 (20.5)	32 (15.5)
60–69	403 (29.6)	230 (31.3)	173 (27.5)	27 (29.7)	82 (24.8)	64 (31.1)
≥70	339 (24.9)	131 (17.8)	208 (33.1)	29 (31.9)	76 (23.0)	103 (50.0)
Comorbidities ^a^	704 (51.7)	336 (45.8)	368 (58.6)	54 (59.3)	163 (49.2)	151 (73.3)
Hypertension	413 (30.3)	192 (26.2)	221 (35.2)	37 (40.7)	95 (28.7)	89 (43.2)
Diabetes	194 (14.2)	97 (13.2)	97 (15.4)	15 (16.5)	45 (13.6)	37 (18.0)
Coronary	104 (7.6)	42 (5.7)	62 (9.9)	8 (8.8)	26 (7.9)	28 (13.6)
Chronic respiratory disease	90 (6.6)	43 (5.9)	47 (7.5)	8 (8.8)	13 (3.9)	26 (12.6)
Chronic digestive disease	73 (5.4)	39 (5.3)	34 (5.4)	2 (2.2)	15 (4.5)	17 (8.3)
Chronic kidney disease	53 (3.9)	21 (2.9)	32 (5.1)	1 (1.1)	18 (5.4)	13 (6.3)
Carcinoma	30 (2.2)	12 (1.6)	18 (2.9)	3 (3.3)	5 (1.5)	10 (4.9)
Others^§^	144 (10.6)	66 (9.0)	78 (12.4)	10 (11.0)	37 (11.2)	31 (15.0)
Fever though clinical course (%)	1179 (86.6)	616 (83.9)	563 (89.6)	77 (84.6)	294 (88.8)	192 (93.2)
Temperature at admission						
<38. 5°C	1048/1179 (88.9)	559/616 (90.7)	489/563 (86.9)	69/77 (89.6)	257/294 (87.4)	163/192 (84.9)
≥38.5°C	123/1179 (10.4)	56/616 (9.1)	67/563 (11.9)	8/77 (10.4)	36/294 (12.2)	23/192 (12.0)
Peak temperature						
<38.5°C	437/1179 (37.1)	245/616 (39.8)	192/563 (34.1)	29/77 (37.7)	100/294 (34.0)	63/192 (32.8)
≥38.5°C	742/1179 (62.9)	371/616 (60.2)	371/563 (65.9)	48/77 (62.3)	194/294 (66.0)	129/192 (67.2)
**Symptoms**						
Cough	1120 (82.2)	594 (80.9)	526 (83.8)	80 (87.9)	282 (85.2)	164 (79.6)
Hypoxia symptoms	953 (70.0)	474 (64.6)	479 (76.3)	62 (68.1)	241 (72.8)	176 (85.4)
Chest tightness	646 (47.4)	348 (47.4)	298 (47.5)	54 (59.3)	145 (43.8)	99 (48.1)
Breath shortness	580 (42.6)	272 (37.1)	308 (49.0)	39 (42.9)	157 (47.4)	112 (54.4)
Dyspnea	481 (35.3)	193 (26.3)	288 (45.9)	24 (26.4)	127 (38.4)	137 (66.5)
Sputum production	709 (52.1)	377 (51.4)	332 (52.9)	52 (57.1)	179 (54.1)	101 (49.0)
Fatigue	675 (49.6)	343 (46.7)	332 (52.9)	48 (52.7)	177 (53.5)	107 (51.9)
Anorexia	610 (44.8)	278 (37.9)	332 (52.9)	37 (40.7)	176 (53.2)	119 (57.8)
Diarrhea	409 (30.0)	231 (31.5)	178 (28.3)	25 (27.5)	92 (27.8)	61 (29.6)
Muscle pain	258 (18.9)	147 (20.0)	111 (17.7)	16 (17.6)	64 (19.3)	31 (15.0)
Nausea	166 (12.2)	100 (13.6)	66 (10.5)	7 (7.7)	38 (11.5)	21 (10.2)
Headache	150 (11.0)	83 (11.3)	67 (10.7)	10 (11.0)	37 (11.2)	20 (9.7)
Vomiting	116 (8.5)	58 (7.9)	58 (9.2)	10 (11.0)	24 (7.3)	24 (11.7)
Sore throat	89 (6.5)	51 (6.9)	38 (6.1)	7 (7.7)	26 (7.9)	5 (2.4)

Notes. All patients who died were included in critical cases.

^a^ Comorbidities listed are not mutually exclusive.

§ Other comorbidities including mental diseases, gynecological diseases, menopausal syndrome, cataracts, hyperthyroidism, hypothyroidism, Alzheimer's disease, paralysis, Parkinson's disease, arthritis, spinal diseases, gout, allergic rhinitis, skin diseases, blood diseases, other cardiovascular and cerebrovascular diseases.

**Fig 3:**
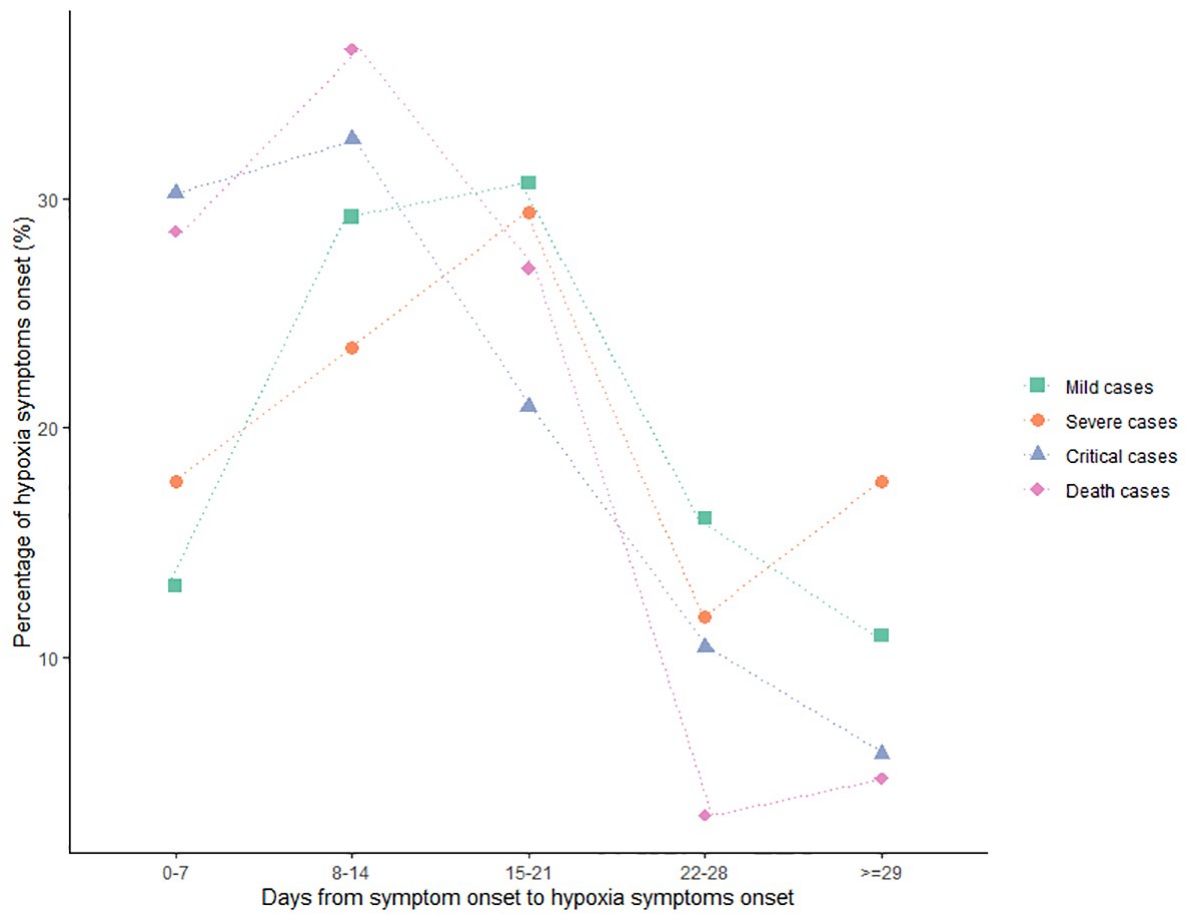
Frequency of hypoxia symptoms onset for patients with different severity of disease by days after onset of symptom (n = 480).

The most common symptoms of COVID-19 were fever (1179, 86.6%) and cough (1120, 82.2%), as shown in **Table 1**. The proportion of fever among hospitalized patients increased with increasing disease severity. The proportion of high fever at admission and the peak temperature ≥38.5°C among fever cases were 10.4% and 62.9%, respectively. Patients with critical illness and fatal illness had the highest proportion of high fever at admission and peak temperature ≥38.5°C, respectively. The proportion of hypoxia symptoms, including chest tightness, shortness of breath and dyspnea, increased with increasing disease severity. Over 66.5% of the patients who died reported dyspnea, followed by shortness of breath (112, 54.4%) and chest tightness (99, 48.1%). The onset of hypoxia symptoms was most common in the second week after symptom onset among patients with a critical and fatal illness, and the onset was most common in the third week among patients with a mild and severe illness (**Fig 3 and** S1 Data).

The patients were admitted to the hospital at a median of 11 days (IQR 7–16) after the onset of symptoms and had a median stay of 18 days (IQR 11–24) in the hospital (**Table 2**). Among them, patients with a fatal illness had the shortest length of stay in the hospital and had a median 8-day (IQR 5–12) length of stay in the ICU. During hospitalization, 1136 (83.4%) patients received oxygen therapy beginning a median of 11 days (IQR 8–17) after symptom onset and continuing for a median of 16 days (IQR 9–23). A total of 244 (17.9%) patients received noninvasive mechanical ventilation treatment beginning a median of 14 days (IQR 10–18) after symptom onset and continuing for a median of 6 days (IQR 3–11). A total of 115 (8.4%) patients received invasive mechanical ventilation beginning a median of 20 days (IQR 16–25) after symptom onset and continuing for a median of 2 days (IQR 1–6). Seven patients with a critical illness, including 2 fatal cases, received ECMO during hospitalization.

**Table 2:** Treatment during hospitalization and clinical course of patients with COVID-2019 in Wuhan, China. Figures are median days (IQR) unless stated otherwise.

Characteristic	All case patients	Patients with mild illness	Patients with severe illness			
			Subtotal	Survivors of severe illness	Survivors of critical illness	Death
**Supplementary oxygen therapy, n (%)**	1136/1362 (83.4)	544/734 (74.1)	592/628 (94.3)	72/91 (79.1)	314/331 (94.9)	206/206 (100)
Time of initiation (days after onset of hypoxia symptoms)	4 (2–8)	4.5 (2–10)	4 (2–7)	3 (2–5)	4 (3–8)	4 (3–5)
Day < = 2^#^	334/480 (69.6)	136/202 (67.3)	198/278 (71.2)	38/49 (77.6)	108/136 (75.0)	52/93 (66.0)
Day >2^#^	146/480 (30.4)	66/202 (31.4)	80/278 (28.8)	11/49 (22.4)	28/136 (25.0)	41/93 (34.0)
Time of initiation (days after onset of symptoms)	11 (8–17)	13 (8–19)	11 (7–15)	12 (8–18)	10 (7–15)	11 (7–15)
Duration of treatment	16 (9–23)	16 (10–23)	16 (9–23)	19 (13–27)	20 (13–25)	10 (6–16)
**Noninvasive Mechanical ventilation, n (%)**	244/1362 (17.9)	-	244/628 (38.9)	-	51/331 (15.4)	193/206 (93.7)
Time of initiation (days after onset of symptoms)	14 (10–18)	-	14 (10–18)	-	12 (10–17)	14 (10–18)
Duration of treatment	6 (3–11)	-	6 (3–11)	-	4 (2–9)	7 (4–11)
**Invasive Mechanical ventilation, n (%)**	115/1362 (8.4)	-	115/628 (18.3)	-	9/331 (2.7)	106/206 (51.5)
Time of initiation (days after onset of symptoms)	20 (16–25)	-	20 (16–25)	-	16 (13–19)	20 (16–25)
Duration of treatment	2 (1–6)	-	2 (1–6)	-	3 (1–4)	2 (1–6)
**ECMO treatment, n (%)**	7/1362 (0.51)	-	7/628 (1.11)	-	3/331 (0.91)	4/206 (1.94)
**Clinical course, n (%)**						
Length of stay in ICU	8 (3–14)	-	8 (3–14)	-	7 (2–15)	8 (5–12)
Length of stay in hospital	18 (11–24)	17 (12–23)	18 (10–26)	21 (15–26)	21 (15–27)	11 (5–18)
From symptom onset to hospital admission	11 (7–16)	12 (7–18)	10 (7–15)	10 (7–16)	10 (7–14)	11 (7–15)
From symptom onset to discharge or death	31 (24–38)	32 (25–39)	30 (22–38)	32 (25–41)	33 (26–39)	23 (17–32)

Notes. All patients who died were critical cases.

^#^ Optimal cut-off values of continuous variables were determined based on the receiver-operating characteristic (ROC) curves.

In the multivariable logistic model (**Table 3**), male sex, age >70 years, shortness of breath, dyspnea and anorexia were independent factors associated with severe illness. Whereas male sex, older age groups, respiratory disease and carcinoma, dyspnea, and no cough symptoms were independent factors associated with a fatal illness. Initiation of oxygen treatment more than 2 days after onset after onset of hypoxia symptoms (OR, 1.92; 95% CI, 1.20 to 3.10) was significantly associated with the risk of death.

**Table 3:** Multivariate analyses* of risk factors associated with severe illness and death in patients with COVID-2019.

Risk factors	OR (95% CI)	*P* value
**Severe illness vs Non-severe illness**		
Male	1.33 (1.05 to 1.67)	0.017
50–59 years of age (vs. 14–49 yrs)	1.29 (0.92 to 1.81)	0.144
60–69 years of age (vs. 14–49 yrs)	1.15 (0.84 to 1.56)	0.382
≥70 years of age (vs. 14–49 yrs)	2.30 (1.66 to 3.21)	<0.001
Peak temperature≥38.5°C	1.26 (0.99 to 1.59)	0.058
Experience Breath shortness	1.38 (1.09 to 1.73)	0.006
Experience dyspnea	2.09 (1.64 to 2.65)	<0.001
Experience anorexia	1.65 (1.31 to 1.08)	<0.001
**Critical illness survivors vs Death**^§^		
Male	1.68 (1.11 to 2.56)	0.014
50–59 years of age (vs. 14–49 yrs)	6.79 (2.83 to 18.49)	<0.001
60–69 years of age (vs. 14–49 yrs)	11.02 (4.88 to 28.74)	<0.001
≥70 years of age (vs. 14–49 yrs)	17.99 (8.09 to 46.42)	<0.001
With respiratory disease	2.48 (1.16 to 5.56)	0.022
With carcinoma	4.11 (1.21 to 16.11)	0.029
No cough	2.46 (1.41 to 4.35)	0.002
Experience dyspnea	2.66 (1.77 to 4.04)	<0.001
Time of oxygen therapy initiation (days after onset of hypoxia symptoms) ^a^		
Day < = 2	Reference	
Day >2	1.92 (1.20 to 3.10)	0.007

*Multivariable logistic regression analyses were performed. Age, hypertension, coronary, respiratory disease, carcinoma were not highly correlated with each other (Spearman’s rank correlation coefficients were calculated; r<0.3 for all), which indicated little evidence for taking account of collinearity in multivariate analyses.

^§^ Only critical cases were included in the analysis.

^a^ Only critical illness patients who reported hypoxia symptoms and received supplementary oxygen treatment were included in the analysis.

Arbidol was the most common drug for antiviral treatment in our study; over 70% of patients were treated with arbidol, with a higher proportion receiving arbidol as a single drug than in combination (**Tables 4–6**). Among noncritical illness patients, LPV/r and oseltamivir were the second and third most common antiviral drugs, and a combination of oseltamivir and arbidol was the most common combination drug treatment (41, 5%) (**Table 4**). Among critical patients, in addition to arbidol, IFN-α was the most common antiviral drug. 57 (10.6%) patients, including 19 (9.2%) patients who died, were treated with a combination of IFN-α and arbidol. 41 (7.6%) critical patients, including 10 (4.9%) patients who died, used IFN-α as a single drug (Table 5). As shown in **Table 6**, after excluding antiviral drugs given after the diagnosis of severe illness, no antiviral treatment was found to be associated with a higher risk of severe illness. Comparing to death, the mortality of critically ill patients who were treated with IFN-a was lower than that of patients not treated with IFN-a (OR, 0.60; 95% CI, 0.39 to 0.91). Critical cases treated with IFN-α had a shorter duration of invasive mechanical ventilation than those not treated with IFNα, but the difference was not statistically significant (*P* value >0.05).

**Table 4:** Combinational antivirus therapy of patients with noncritical illness.

Antivirus treatment	Patients with mild cases (n = 734)	Patients with severe cases^#^ (n = 91)	Total
1	439 (59.8)	56 (61.5)	495 (60)
2	39 (5.3)	4 (4.4)	43 (5.2)
1+3	38 (5.2)	3 (3.3)	41 (5)
3	29 (4)	1 (1.1)	30 (3.6)
1+5	24 (3.3)	3 (3.3)	27 (3.3)
1+2	15 (2)	4 (4.4)	19 (2.3)
1+6	12 (1.6)	1 (1.1)	13 (1.6)
6	9 (1.2)	4 (4.4)	13 (1.6)
5	8 (1.1)	2 (2.2)	10 (1.2)
4	4 (0.5)	1 (1.1)	5 (0.6)
3+5	3 (0.4)	0 (0)	3 (0.4)
1+5+6	2 (0.3)	0 (0)	2 (0.2)
1+4	1 (0.1)	1 (1.1)	2 (0.2)
3+6	1 (0.1)	1 (1.1)	2 (0.2)
2+5	2 (0.3)	0 (0)	2 (0.2)
1+3+6	0 (0)	1 (1.1)	1 (0.1)
1+3+5	0 (0)	1 (1.1)	1 (0.1)
2+6	0 (0)	1 (1.1)	1 (0.1)
2+4	1 (0.1)	0 (0)	1 (0.1)
2+3	1 (0.1)	0 (0)	1 (0.1)

**Note.** Arbidol = 1; LPV/r = 2; Oseltamivir = 3; CQ or HCQ = 4; IFN-α = 5; Ribavirin = 6

^#^Only antivirus drugs given before diagnosis of severe illness were counted, antivirus treatment before admission was counted for patients with severe illness when admission, critical cases were excluded.

**Table 5:** Combinational antivirus therapy of patients with critical illness.

Antivirus treatment	Critical illness survivors^§^ (n = 331)	Death^§^ (n = 206)	Total
1	145 (43.8)	69 (31.6)	210 (39.1)
1+5	38 (11.5)	19 (9.2)	57 (10.6)
5	31 (9.4)	10 (4.9)	41 (7.6)
2	13 (3.9)	11 (5.3)	24 (4.5)
1+2	11 (3.3)	5 (2.4)	16 (3)
1+4	11 (3.3)	3 (1.5)	14 (2.6)
1+2+5	10 (3)	3 (1.5)	13 (2.4)
1+6	4 (1.2)	4 (3.9)	12 (2.2)
1+3	7 (2.1)	3 (1.5)	10 (1.9)
6	9 (0.6)	2 (2.4)	7 (1.3)
5+6	1 (0.3)	5 (2.4)	6 (1.1)
3+5	3 (0.9)	1 (0.5)	4 (0.7)
2+5	3 (0.9)	1 (0.5)	4 (0.7)
1+2+6	1 (0.3)	3 (1.5)	4 (0.7)
3	2 (0.6)	1 (0.5)	3 (0.6)
1+3+5	3 (0.9)	0 (0)	3 (0.6)
2+5+6	0 (0)	2 (1)	2 (0.4)
2+4	1 (0.3)	1 (0.5)	2 (0.4)
1+5+6	2 (0.6)	0 (0)	2 (0.4)
1+4+6	2 (0.6)	0 (0)	2 (0.4)
1+2+5+6	1 (0.3)	1 (0.5)	2 (0.4)
4	1 (0.3)	0 (0)	1 (0.2)
2+3	1 (0.3)	0 (0)	1 (0.2)
2+3+5	1 (0.3)	0 (0)	1 (0.2)
1+3+4	1 (0.3)	0 (0)	1 (0.2)
1+2+4	0 (0)	1 (0.5)	1 (0.2)
1+2+3	1 (0.3)	0 (0)	1 (0.2)

**Note.** Arbidol = 1; LPV/r = 2; Oseltamivir = 3; CQ or HCQ = 4; IFN-α = 5; Ribavirin = 6

^§^Only antivirus drugs given during critical illness course are counted.

**Table 6:** Multivariate analyses* of antivirus treatment associated with severe illness and death in patients with COVID-2019.

Antivirus treatment	No. (%) of patients				OR (95% CI), *P*	
	Patients with mild cases (n = 734)	Severe illness survivors^#^ (n = 91)	Critical illness survivors^§^ (n = 331)	Death^§^ (n = 206)	Mild vs severe	Critical vs death
Arbidol	531 (72.3)	70 (76.9)	237 (71.6)	111 (53.9)	1.08 (0.59 to 2.09), 0.807	0.72 (0.42 to 1.24), 0.232
LPV/r	58 (6.1)	9 (9.9)	43 (13.0)	28 (13.6)	1.26 (0.55 to 2.60), 0.556	1.15 (0.60 to 2.18), 0.665
Oseltamivir	72 (9.8)	7 (7.7)	19 (5.7)	5 (2.4)	0.66 (0.27 to 1.45), 0.324	0.40 (0.12 to 1.14), 0.105
IFN-α	39 (5.3)	6 (6.6)	93 (28.1)	42 (20.4)	1.22 (0.44 to 2.90), 0.679	0.60 (0.39 to 0.91), 0.017
Ribavirin	24 (3.3)	8 (8.8)	20 (6.0)	17 (8.3)	1.23 (0.83 to 1.82) 0.309	1.32 (0.78 to 2.22), 0.290
CQ or HCQ	6 (0.8)	2 (2.2)	16 (4.8)	5 (2.4)	2.59 (0.37 to 11.87), 0.256	0.45 (0.13 to 1.31), 0.166

*Adjusted for valuables listed in Table 3.

^#^ Only antivirus drugs given before diagnosis of severe illness were counted, antivirus treatment before admission was counted for patients with severe illness when admission, critical cases were excluded.

^§^Only antivirus drugs given during critical illness course are counted.

## Discussion

Initiation of oxygen treatment less than 2 days after onset after onset of hypoxia symptoms and the use of IFN-a among critically ill patients were significantly associated with lower risk of COVID-19 mortality. Our study highlighted the importance of early oxygen therapy and lend support to the potentially beneficial effects of IFNα on critical illness.

In this study, the proportion of male patients, older age and comorbidities increased with increasing disease severity. This finding was consistent with results from many other studies [8–11]. Older patients with comorbidities were more likely to develop severe and fatal illnesses than younger patients in this study. More attention should be paid to the potential effect of COVID-19 on the original comorbidities of older patients and the protection and prevention thereof in the treatment of COVID-19 [1,12,13]. Respiratory disease and carcinoma were identified as risk factors for fatal illness in this study. Severe COVID-19 may cause damage to multiple organs, especially to the respiratory tract, and underlying chronic respiratory disease are serious comorbid conditions that may affect the survival rate when treating critical illness [14].

Similar to the other two novel coronaviruses causing lethal zoonotic diseases in humans, namely SARS and Middle East respiratory syndrome (MERS), fever was the dominant symptom of COVID-19. In our study, 86.6% of the patients had a fever, which was lower than proportion in patients with SARS or MERS [15,16]. Cough, the second most common symptom of COVID-19, occurred in critical illness survivors more frequently than in those that died. This may be due to the rapid disease progression in the patients that died. Alternatively, these patients may have delayed their visit to the hospital due to a lack of typical respiratory symptoms. In our study, patients with a cough had a shorter time to seek medical treatment after the onset of symptoms than those without a cough, but this difference was not significant (*P* value >0.05). The most common gastrointestinal symptom in our study was anorexia, followed by diarrhea, nausea and vomiting. Our multivariable analyses suggested that anorexia was associated with severe COVID-19 illness. This result supports a previous multicenter study, which indicated that digestive symptoms became more pronounced as the severity of the disease increased [17]. For patients with digestive symptoms, it may be necessary to pay attention closely in advance rather than waiting for respiratory symptoms to emerge.

Generally, the occurrence of dyspnea in patients with COVID-19 is less frequent than in patients with MERS [18,19] and SARS [20–22]. However, differences in the frequency of these symptoms among some recent studies are considerable. In a prospective study conducted at the beginning of the outbreak in Wuhan, dyspnea occurred in 55% of inpatients [1]. One study extracted the data on 1,099 patients from 552 hospitals in 31 provinces and indicated that only 18.6% of patients experienced shortness of breath [8]. In a retrospective single-center study that included 155 patients in a hospital in Wuhan, the proportions of patients with chest distress, shortness of breath and dyspnea were 39.4%, 32.3% and 2.4%, respectively [9]. In addition to the different distributions of mild and severe diseases, these inconsistent clinical research results may also be due to the various ways to express feeling breathless, which can be described as shortness of breath, dyspnea and chest tightness in the medical records. It is likely patients under different hypoxic conditions might express the feeling of breathlessness inaccurately and consistently. After grouping these three symptoms as hypoxia symptoms, 70.0% of patients had hypoxia symptoms, and most of them received oxygen therapy. In addition, the onset of hypoxia symptoms was most common in the second to third week after symptom onset, and patients with critical illness and who died experienced these symptoms earlier than those with mild and severe illness. The results suggested that both clinicians and isolated patients at home need to focus on and maintain their vigilance even if the first week after disease onset has passed [1].

Early recognition of patients with worsening respiratory function while on conventional oxygen therapies is important to ensure the timely and safe escalation of respiratory support [6]. Hypoxia symptoms as an indicator of hypoxia injury were recognized to be one of the most important hallmarks of COVID-19 coronavirus infection and can easily be identified by patients themselves [23]. The association between oxygen therapy initiation after the onset of hypoxia symptoms and the outcome of the disease was analyzed in our study. The results indicated that oxygen therapy that was started more than 2 days after hypoxia symptom onset significantly increased the risk of death outcomes. As early initiation of oxygen therapy may reduce disease mortality for patients with COVID-19, our findings highlighted the importance of early oxygen therapy after the onset of hypoxia symptoms.

In our study, IFN-a was the second most common antiviral drug used in patients with critical illness, and it was mainly used in combination with oseltamivir. After adjusting for other risk factors, an inverse association was observed between IFN-a use and the mortality among patients with critical illness. In the context of emerging viral infections, IFN-α is often evaluated before specific treatments are developed due to its unspecific antiviral effects [24,25]. IFN-α treatment has been studied in MERS-CoV and SARS-CoV infections in numerous experiments, both in vitro and in vivo, and in combination or with other antiviral drugs or not [26,27]. Recently, an in vitro study indicated that SARS-CoV-2 is much more sensitive to IFNα pretreatment than SARS-CoV at the molecular level [27,28]. The Chinese guidelines for the treatment of COVID-19 recommend IFNα use in combination with ribavirin. However, there is currently no clear consensus on the role of IFNα in COVID-19 development or progression. Nevertheless, our results lend support to the potentially beneficial effects of IFNα in patients with critical illness. Given these preliminary data, further studies are needed to investigate whether IFNα has an effect in preventing complications associated with COVID-19 critical illness in a large population.

### Limitations of the study

Our study has limitations. First, this retrospective observational study was performed at a designed hospital that was urgently reconstructed and has been assigned by the Chinese government for severely or critically ill patients with COVID-19. The proportion of severe and critical diseases was high in our study. To avoid selection bias, we compared risk factors for patients with critical cases and patients who died for death-related risk factors instead of comparing patients with fatal cases to patients with nonfatal cases. Second, because there is currently no clear consensus on the role of combinational antiviral therapy in the development and progression of COVID-19, while combinational antiviral therapy for severe patients is exploratory, especially for critical patients. The effect of combinational antiviral therapy was not considered in our multivariate analyses, although it may help to provide better clinical benefits. In addition, our findings should be interpreted with caution because of the retrospective study design and selection bias, although the number of cases in our study allowed statistical power to assess potential independent risk factors associated with severe COVID-19 illness.

In conclusion, the main differences in the clinical features of patients with different severities of COVID-19 were described and the risk factors to expand the current recommendations for high-risk groups were explored in this study. Our findings highlighted the importance of early oxygen therapy after the onset of hypoxia symptoms. In addition, our results lend support to the potentially beneficial effects of IFNα on critical illness.

## Supporting information

S1 STROBE Checklist (DOCX)

S1 Data Excel spreadsheet containing, in separate sheets, the underlying numerical data and statistical analysis for Figures. (XLSX)
